# Absence of anti–rabphilin-3A antibodies in children and young adults with idiopathic central diabetes insipidus: a potential clue to elucidating a tumor etiology

**DOI:** 10.1007/s42000-023-00484-0

**Published:** 2023-09-11

**Authors:** Haruki Fujisawa, Takako Takeuchi, Akira Ishii, Jun Muto, Hotaka Kamasaki, Atsushi Suzuki, Yoshihisa Sugimura

**Affiliations:** 1 Department of Endocrinology, Diabetes and Metabolism, Fujita Health University, 1-98 Dengakugakubo, Kutsukake-Cho, Toyoake, Aichi 470-1192 Japan; 2 Department of Pediatrics, Sapporo Medical University School of Medicine, Sapporo, 060-8556 Japan; 3 Department of Neurosurgery, Fujita Health University, Toyoake, Aichi 470-1192 Japan

**Keywords:** AVP, Germ cell tumor, LINH, Pituitary, PLAP

## Abstract

**Background:**

Central diabetes insipidus (CDI) is a rare condition caused by various underlying diseases, including neoplasms, autoimmune diseases, and infiltrative diseases. Differentiating between CDI etiologies is difficult. What has initially been classified as “idiopathic” central diabetes insipidus might in fact underlie various pathogenic mechanisms that are less understood to date and/or are not obvious at initial presentation. Therefore, even if idiopathic CDI is diagnosed at the time of onset, it is common for tumors such as germinoma to develop during surveillance. Crucially, a delayed diagnosis of germinoma may be associated with a worse prognosis. Recently, the presence of anti–rabphilin-3A antibodies has been found to be a highly sensitive and specific marker of lymphocytic infundibuloneurohypophysitis, an autoimmune-mediated CDI.

**Case presentation:**

We herein present two cases, namely, a 13-year-old boy (patient 1) and a 19-year-old young man (patient 2) who were diagnosed with idiopathic CDI. In both patients, panhypopituitarism developed. Magnetic resonance imaging revealed pituitary stalk thickening and pituitary swelling approximately 1 1/2 years after the onset of CDI. Western blotting did not reveal the presence of anti-rabphilin-3A antibodies in serum in either patient, suggesting that autoimmune mechanisms might not be involved. Both patients were subsequently diagnosed with germinoma on pathological examination. They received chemotherapy, followed by radiation therapy. Notably, testosterone and insulin-like growth factor-1 levels normalized, and libido and beard growth recovered after chemoradiotherapy in patient 2.

**Conclusion:**

Our data suggest that the absence of anti-rabphilin-3A antibodies in young patients clinically diagnosed with idiopathic CDI may increase the probability of the development of non-lymphocytic lesions, including germinoma. We thus recommend a more attentive approach at the onset of these diseases.

**Supplementary Information:**

The online version contains supplementary material available at 10.1007/s42000-023-00484-0.

## Introduction

Central diabetes insipidus (CDI) is caused by the destruction or degeneration of neurons originating in the supraoptic and paraventricular nuclei of the hypothalamus, leading to a deficiency in the secretion of arginine vasopressin (AVP), polyuria, and polydipsia [1, 2]. The underlying etiology of CDI includes tumors (such as germinomas, craniopharyngiomas, and large pituitary adenomas, whether functional or not), infiltrative diseases (such as Langerhans cell histiocytosis), autoimmune diseases, neurosurgery, trauma, malformations, and, in rare cases, genetic defects in AVP synthesis [2–5]. In some cases, at initial presentation, no underlying etiology can be identified even by the performance of careful diagnostic work-up, including neuroimaging, and the CDI of these cases is therefore considered idiopathic [6]. However, when a preliminary diagnosis of idiopathic CDI is made, concerns remain that some diagnoses, such as Langerhans cell histiocytosis, germinoma, local inflammatory processes, or autoimmune processes, may not be evident at the time of diagnosis of CDI [7]. Di Iorgi et al. reported that only 24 of 85 children or young adult patients with CDI received an etiologic diagnosis at the time of first presentation, and seven and four patients were diagnosed with germinoma and Langerhans cell histiocytosis, respectively, within 2.5 years of a diagnosis of CDI [8]. They also reported that the etiologies of 51.3% of children or young adult patients with CDI were inflammatory/autoimmune processes; idiopathic CDI accounted for only 4% of patients with CDI [8].

Intracranial germ cell tumors (GCTs) are rare central nervous system tumors that are predominantly found in children and young adults. They usually occur in pineal (51%) and suprasellar (30%) regions, both of which are midline structures [9]. Intracranial GCTs are divided into germinomas and nongerminomatous GCTs. Nongerminomatous GCTs contain nongerminomatous elements, which confer a worse prognosis [10]. Symptoms of suprasellar GCTs include anterior pituitary dysfunction, mainly growth hormone deficiency, CDI, and visual defects reflecting the anatomical location of the tumor [11]. Patients with suprasellar GCT occasionally exhibit normal or slightly abnormal neuroimaging findings, including a loss of bright signal intensity of the posterior pituitary on T1-weighted magnetic resonance imaging (MRI) or pituitary stalk thickening at presentation with endocrinopathies [12]. Therefore, many patients experience a long symptomatic period before the diagnosis of a GCT [13, 14]. Furthermore, patients with an intracranial GCT and symptoms of ≧ 6 months’ duration are more likely to have disseminated disease [15]. Therefore, early detection and treatment are important in order to minimize morbidity and mortality [16].

Lymphocytic infundibuloneurohypophysitis (LINH) accounts for a substantial subset of autoimmune CDI cases and is characterized by lymphocytic inflammation of the posterior pituitary and infundibular stalk [2, 4, 17, 18]. Clinically, a differential diagnosis of LINH and other pituitary diseases that cause CDI, such as sellar or suprasellar tumors, including intracranial GCTs and Langerhans cell histiocytosis, can be difficult because of a similar clinical presentation and radiographic appearance [19]. We have previously shown that the presence of anti-rabphilin-3A antibodies is a highly sensitive and specific diagnostic marker for LINH [20]. In this report [20], all 34 biopsy-proven samples from sellar/suprasellar masses, including five germinomas, were negative for anti-rabphilin-3A antibodies, yielding a specificity of 100% for distinguishing sellar/suprasellar masses. Therefore, the presence of anti-rabphilin-3A antibodies would almost entirely exclude the possibility of intracranial GCTs in patients with CDI.

We aimed to confirm the usefulness of measuring anti-rabphilin-3A antibodies in the diagnosis of non-lymphocytic lesions, including germinoma with CDI without an obvious tumor in sellar or suprasellar regions.

## Methods

### Measurement of anti-rabphilin-3A antibodies by western blotting

A vector containing the full-length human rabphilin-3A gene was transfected into HEK293FT cells to produce recombinant human rabphilin-3A protein. The vector was designed to fuse a V5 tag to the encoded rabphilin-3A protein. The rabphilin-3A gene was inserted into the N-terminus of the V5 tag (pcDNA3.1-rabphilin 3A-V5-His). V5 expression depends on the start codon of rabphilin-3A. The expression of recombinant rabphilin-3A protein was detected using an anti-V5 antibody. As a control, the same vector but without the rabphilin-3A gene was transfected into HEK293FT cells. Anti-rabphilin-3A antibodies in serum were detected by western blotting using recombinant human rabphilin-3A protein lysate as an antigen source and serum as the primary antibody. A protein band of 76 kDa that appeared in the lysate of cells transfected with rabphilin-3A protein but not in that of control cells was considered to be positive for anti-rabphilin-3A antibodies, as reported previously [20].

### Immunocytochemistry

A vector containing the full-length human rabphilin-3A gene (pcDNA3.1-rabphilin 3A-V5-His) was used. The vector was transfected into COS-7 cells using Lipofectamine® 2000 reagent (Thermo Fisher Scientific, Waltham, MA, USA). We used untransfected COS-7 cells as negative controls. After 24-h incubation, the cells were fixed with 4% paraformaldehyde for 20 min at room temperature. After washing with phosphate buffered saline, the cells were incubated with 0.3% Triton X-100 followed by incubation with both patient sera (1:50) and anti-V5 antibodies (1:200, Invitrogen, Waltham, MA, USA) as primary antibodies. To establish the identification of rabphilin-3A by patient sera, cells were stained with Alexa Fluor 488-conjugated anti-human IgG and Alexa Fluor 594-conjugated anti-mouse IgG as secondary antibodies. Colocalization was observed with a fluorescence microscope (BZ-X800; Keyence, Osaka, Japan) [18].

### Case presentation

#### Patient 1

A boy, aged 13 years and 11 months, was admitted to Sapporo Medical University Hospital, Sapporo, Japan, in December 2015 due to persistent thirst, polydipsia causing appetite loss, and polyuria for 2 months. His growth curve and puberty progression were normal. On physical examination, his height was 174.5 cm (+ 1.74 SD) and weight was 54.5 kg (+ 0.23 SD). He had dry lips and oral mucosa; the remainder of the examination was unremarkable. The patient’s visual field was normal and his genital Tanner stage was IV. Testicular volumes, measured using an orchidometer, were approximately 10 mL. The patient’s water intake was 5550 mL/day and urine volume was 3670 mL/day.

**Fig. 1:**
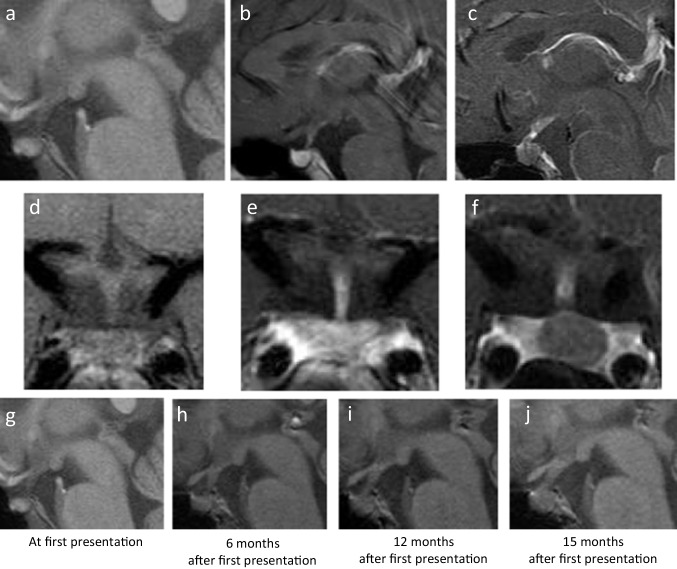
MRI images of pituitary lesion in patient 1. Sagittal (**a**) and coronal (**d**) plain T1-weighted images at first presentation. Sagittal (**b**) and coronal (**e**) gadolinium-enhanced T1-weighted images at first presentation. Sagittal (**c**) and coronal (**f**) gadolinium-enhanced T1-weighted images 15 months after first presentation. Serial sagittal plain T1-weighted images (**g**–**j**). At first presentation (**g**), and 6 (**h**), 12 (**i**), and 15 (**j**) months after first presentation. MRI, magnetic resonance imaging

Laboratory examination revealed low spot urine osmolality (98 mmOsm/kg, reference range: 50–1300) and normal serum osmolality (282 mmOsm/kg, reference range: 275–290). No abnormalities were noted in liver function, renal function, serum electrolytes, and in a baseline assessment of pituitary hormones (Table 1). We performed a 4.5 h-water deprivation test, which demonstrated insufficient elevation of urinal osmolality (from 84 to 152 mmOsm/kg) and a clear response to the subcutaneous injection of 5.0 U AVP (from 152 to 529 mmOsm/kg). Plain MRI revealed a loss of hyperintense area in T1-weighted images (Fig. 1a). The pituitary stalk was not thickened (diameter 2.0 mm; Fig. 1a and d). The pituitary lobe was homogeneously enhanced by gadolinium-based contrast agents (Fig. 1b and e). Serum human β-chorionic gonadotropin (β-hCG) was not elevated. The patient was diagnosed with idiopathic CDI and oral desmopressin acetate hydrate (60 µg in the morning and 60 µg at night) was administered, resulting in the remission of the persistent thirst, polydipsia, and polyuria (water intake and urine volume were around 1–1.5 L/day).

**Table 1:** Endocrinological findings and tumor markers for case 1

Variable	Reference range	On first admission (13 years 11 months)	On second admission (15 years 2 months)
Blood			
ACTH (pg/mL)	7.2–63.3	31.4	13.4
Cortisol (µg/dL)	7.07–19.6	7.7	6.35
TSH (µU/mL)	0.25–4.5	2.53	2.36
Free T4 (ng/dL)	0.8–2.0	1.24	0.68
IGF-1 (ng/mL)	13 y.o. boy; 133–579	232	108
	15 y.o. boy; 141–552		
LH (mU/mL)	1.27–9.57	1.68	0.56
FSH (mU/mL)	1.18–15.82	2.72	0.44
Testosterone (ng/mL)	2.84–7.99	6.24	0.29
Prolactin (ng/mL)	4.3–13.7	15.02	46.45
AFP (ng/mL)	< 8.5		4.6
β-hCG (ng/mL)	< 0.1	< 0.1	< 0.1
IgG4 (mg/dL)	11–121		23.5
ACE	4.8–105.0		20.9
CSF			
AFP (ng/mL)			< 0.4
β-hCG (ng/mL)			0.3
PLAP (pg/mL)	< 30		1080

*ACE* angiotensin-converting enzyme, *ACTH* adrenocorticotropic hormone, *AFP* alpha-fetoprotein, *β-hCG* β-human chorionic gonadotropin, *CSF* cerebrospinal fluid, *FSH* follicle-stimulating hormone, *IGF-1* insulin-like growth factor 1, *LH* luteinizing hormone, *PLAP* placental alkaline phosphatase, *TSH* thyroid-stimulating hormone, *y*.*o*. year old

The patient visited our hospital every 3 to 6 months for the monitoring of anterior pituitary functions and for a brain MRI. He recovered his appetite and 6 months after the first presentation, his weight had increased to 60.1 kg. However, in the subsequent 3 months, he began to experience severe fatigue and appetite loss. His thyroid and gonadal functions gradually deteriorated and his serum prolactin (PRL) level became elevated. MRI also revealed gradual thickening of the pituitary stalk (Fig. 1g–j). Therefore, readmission for the evaluation of pituitary function and the etiology of the pituitary lesion was planned. Hormone replacement was not started before the readmission.

**Fig. 2:**
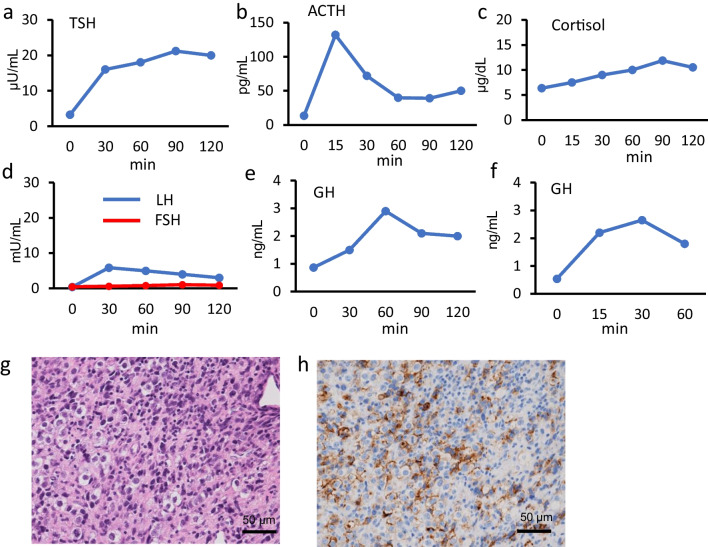
Endocrinological stimulation test results 15 months after initial presentation and biopsy specimens from pituitary lesion in patient 1. Response of TSH to intravenous injection of TRH (**a**). Response of ACTH (**b**) and cortisol (**c**) to intravenous injection of CRH. Response of LH and FSH to intravenous injection of LHRH (**d**). Response of GH to intravenous arginine (**e**) and GHRP2 (**f**). Hematoxylin and eosin staining (**g**) and immunohistochemistry for PLAP (**h**) of biopsy specimens from pituitary lesion. ACTH, adrenocorticotropic hormone; CRH, corticotropin-releasing hormone; FSH, follicle-stimulating hormone; GH, growth hormone; GHRP2, growth hormone-releasing peptide-2; LH, luteinizing hormone; LHRH, luteinizing hormone-releasing hormone; PLAP, placental alkaline phosphatase; TRH, thyrotropin-releasing hormone; TSH, thyroid-stimulating hormone

Fifteen months after initial presentation, at the age of 15 years and 2 months, the patient was readmitted for further evaluation. His height had increased to 177.0 cm (+ 1.54 SD), while his weight had decreased to 57.4 kg (− 0.08 SD). Physical examination revealed a decrease in his testicular volume, measured as approximately 6 mL, and a loss of axillary hair (pubic hair was intact). The dose of oral desmopressin administered was not changed because the patient’s intake and output were unchanged after starting oral desmopressin and he did not have persistent thirst, polyuria, or polydipsia. A blood examination revealed central hypothyroidism (low free T4 of 0.68 ng/dL [reference range 0.8–2.0 ng/dL]), with an inappropriately normal thyroid stimulating hormone (TSH) level of 2.36 µU/mL (reference range 0.25–4.5 µU/mL), decreased serum insulin-like growth factor (IGF)-1, luteinizing hormone (LH), follicle stimulating hormone (FSH), and testosterone, and increased PRL (Table 1). A thyrotropin-releasing hormone stimulation test revealed a delayed response of TSH (Fig. 2a). A corticotropin-releasing hormone (CRH) stimulation test revealed that the cortisol response to CRH was low, while the adrenocorticotropic hormone (ACTH) response was high, suggesting suprapituitary lesions (Fig. 2b and c). Luteinizing hormone-releasing hormone (LHRH), arginine, and growth hormone-releasing peptide-2 (GHRP2) stimulation tests also revealed gonadotropin and growth hormone (GH) deficiencies (Fig. 2d–f). Serum and cerebrospinal fluid (CSF), β-hCG, and alpha-fetoprotein (AFP) were not elevated. However, CSF placental alkaline phosphatase (PLAP) was significantly elevated (1080 ng/mL: reference range < 30 ng/mL [21]) (Table 1). A hypoenhanced area in the pituitary stalk extending to the posterior pituitary, which was not present at first admission, was detected by gadolinium-enhanced MRI (Fig. 1c and f).

After starting supplementation with levothyroxine and hydrocortisone, a posterior pituitary biopsy was performed. The obtained biopsy specimen showed dense inflammatory infiltrate of small lymphocytes and scattered large, atypical cells with abundant clear cytoplasm and round nuclei. Such atypical cells stained positive for PLAP by immunohistochemistry (Fig. 2g and h). According to these pathological findings, a diagnosis of germinoma was established. The patient underwent four courses of combined chemotherapy (etoposide and cisplatin) and 24 Gy/16 Fr of whole ventricle radiation therapy. Five months after the completion of chemoradiotherapy, the low intensity area in the pituitary stalk and posterior pituitary had disappeared. Supplementation with desmopressin, levothyroxine, and hydrocortisone was continued.

#### Patient 2

**Fig. 3:**
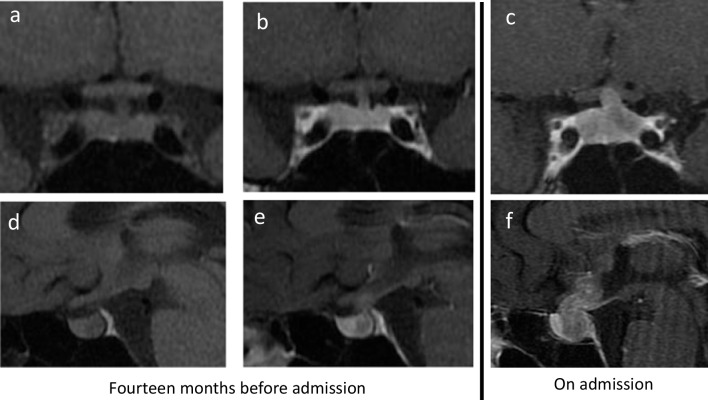
MRI images of pituitary lesion in patient 2. Coronal (**a**) and sagittal (**d**) plain T1-weighted images 14 months before admission to our hospital. Coronal (**b**) and sagittal (**e**) gadolinium-enhanced T1-weighted images 14 months before admission to our hospital. Coronal (**c**) and sagittal (**f**) gadolinium-enhanced T1-weighted images at the time of admission to our hospital. MRI, magnetic resonance imaging

A 21-year-old man was admitted to Fujita Health University Hospital, Toyoake, Aichi, Japan, because of malaise and orthostatic syncope in July 2019. One and half years prior to admission, increased thirst, fluid consumption (about 4 L/day), and frequent urination had suddenly developed. Fourteen months before admission, the patient was diagnosed with CDI and a prescription of oral desmopressin was started at another hospital. At the time, his endocrinological data were almost normal except for slightly decreased IGF-1 and a low plasma AVP concentration (Table 2). A T1-weighted image showed the absence of a high-intensity signal in the posterior lobe (Fig. 3a and d). In addition, a gadolinium-enhanced MRI revealed an ill-defined hypoenhanced area relative to the surrounding tissues in the pituitary gland (Fig. 3b and e). However, neither a follow-up endocrinological test nor an MRI was performed for more than 1 year. One month before admission, malaise and orthostatic syncope had developed and the patient subsequently visited another hospital. Gadolinium-enhanced MRI revealed a weakly enhancing mass in the sellar and suprasellar regions (Fig. 3c and f) and the patient was referred to our hospital. He also had a reduced libido and slow beard growth for around 6 months.

**Table 2:** Endocrinological findings and tumor markers for case 2

Variable	Reference range	14 months before admission	On admission
Blood			
ACTH (pg/mL)	7.2–63.3	32.8	10.4
Cortisol (µg/dL)	6.2–18	12.0	1.390
TSH (µU/mL)	0.50–5.00	0.837	1.17
Free T3 (pg/mL)	2.51–4.16	3.2	2.25
Free T4 (ng/dL)	0.83–1.77	1.8	0.63
IGF-1 (ng/mL)	142–470	137	60
LH (mU/mL)	2.2–8.4	6.1	< 0.09
FSH (mU/mL)	1.8–12	2.3	0.11
Testosterone (ng/mL)	1.31–8.71	8.32	< 0.03
Prolactin (ng/mL)	3.5–19.4		55.7
AVP (pg/mL)		< 0.4	< 0.4
AFP (ng/mL)	0–10.0		2.6
β-hCG (ng/mL)	0–0.1		< 0.1
IgG4 (mg/dL)	11–121		12.5
ACE (U/L)	8.3–21.4		14.1
Urine			
Cortisol (µg/day)	11.2–80.3		11.7
CSF			
AFP (ng/mL)			< 2.0
hCG (mU/mL)			< 2.0

*ACE* angiotensin-converting enzyme, *ACTH* adrenocorticotropic hormone, *AFP* alpha-fetoprotein, *AVP* arginine vasopressin, *β-hCG* β-human chorionic gonadotropin, *CSF* cerebrospinal fluid, *FSH* follicle-stimulating hormone, *hCG* human chorionic gonadotropin, *IGF-1* insulin-like growth factor 1, *LH* luteinizing hormone, *PLAP* placental alkaline phosphatase, *TSH* thyroid-stimulating hormone

**Fig. 4:**
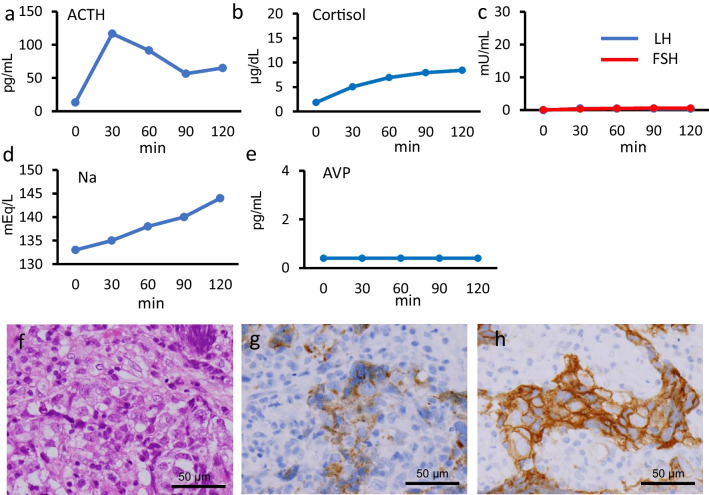
Endocrinological stimulation test results and biopsy specimens from pituitary lesion in patient 2. Response of ACTH (**a**) and cortisol (**b**) to intravenous injection of CRH. Response of LH and FSH to intravenous injection of LHRH (**c**). Serum sodium (**d**) and plasma AVP concentrations during hypertonic saline test. Hematoxylin and eosin staining (**f**) and immunohistochemistry for PLAP (**g**) and c-kit (**h**) in biopsy specimens from pituitary lesion. ACTH, adrenocorticotropic hormone; AVP, arginine vasopressin; CRH, corticotropin-releasing hormone; FSH, follicle-stimulating hormone; LH, luteinizing hormone; LHRH, luteinizing hormone-releasing hormone; PLAP, placental alkaline phosphatase

On examination, the patient was alert and his blood pressure was 125/63 mmHg; other vital signs were normal. His height was 170 cm and his body weight was 56 kg. He had a sparse beard, but axillary and pubic hairs were intact. A white blood cell count was normal but showed a high percentage of eosinophils (9.0%: reference range 0.4–8.6%). Serum sodium concentration was slightly low (133 mEq/L: reference range 138–145 mEq/L). Other serum electrolytes and liver and renal functions were normal. A baseline assessment of pituitary hormones revealed central hypothyroidism (low free T4 of 0.63 ng/dL with an inappropriately normal TSH of 1.17 µU/mL), decreased cortisol with normal ACTH, decreased serum IGF-1, LH, FSH, and testosterone, and increased PRL (Table 2). A CRH stimulation test revealed that the cortisol response to CRH was low, while the ACTH response was high. This suggested that the lesions in this patient mainly existed at the suprapituitary level taking into account the increased PRL concentration (Fig. 4a and b). The LH and FSH responses to LHRH were low (Fig. 4c). A hypertonic saline test showed no AVP response to an increased serum sodium concentration, which is compatible with complete CDI (Fig. 4d and e). Serum and CSF β-hCG and AFP levels were not elevated (Table 2). A visual field test was normal.

A hydrocortisone and levothyroxine prescription was started and transsphenoidal biopsies were performed. Histological findings were similar to those of patient 1 and the patient was also diagnosed with a pure germinoma (Fig. 4f–h).

**Fig. 5:**
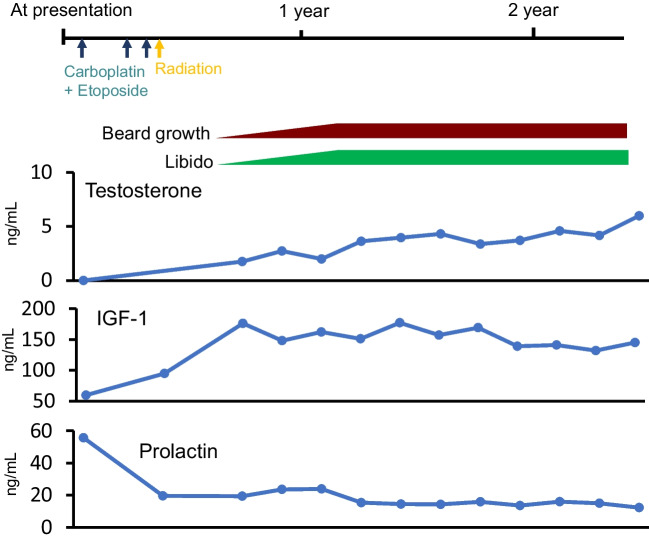
Temporal profile of symptoms, serum testosterone, IGF-1, and prolactin levels in patient 2. IGF-1, insulin-like growth factor

Subsequently, the patient underwent three cycles of chemotherapy (carboplatin and etoposide), followed by radiation therapy consisting of 24 Gy/12 Fr to the whole ventricle and a 12 Gy/6 Fr additional boost to the tumor. Approximately 3 months after the completion of chemoradiotherapy, the patient’s libido and beard growth began to recover. In parallel, serum testosterone and IGF-1 increased, while the serum PRL level decreased (Fig. 5). However, supplementation with desmopressin, levothyroxine, and hydrocortisone could not be discontinued. The patient has had no clinical or imaging evidence of recurrent disease for 2 years since the completion of chemoradiotherapy.

### Anti-rabphilin-3A antibodies

**Fig. 6:**
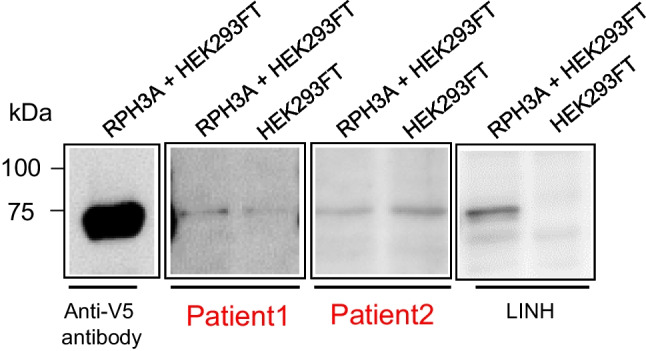
Detection of anti-rabphilin-3A antibodies by western blotting. Recombinant full-length human rabphilin-3A expressed in HEK293FT cells (RPH3A + HEK293FT, left lane in each case) or negative control (HEK293FT, right lane in each case) was probed with sera from patients 1 and 2, and a patient with LINH. Recombinant full-length human rabphilin-3A expressed in HEK293FT cells was also probed with an anti-V5 antibody as a positive control (anti-V5 antibody) in the first lane from the left. Serum from a patient with LINH reacted with only recombinant full-length human rabphilin-3A (RPH3A + HEK293FT lane), but sera from patients 1 and 2 also reacted with a negative control (HEK293 FT lane). LINH, lymphocytic infundibuloneurohypophysitis

Serum samples for the measurement of anti-rabphilin-3A antibodies were obtained about 1 1/2 years after the onset of CDI in both cases. Anti-rabphilin-3A antibodies as evaluated by western blotting were not detected in both cases (Fig. 6). We further performed immunocytochemical experiments to exclude the influence of conformational structure under nonreducing conditions [20]. The negative test results from western blotting using anti-rabphilin-3A antibodies were confirmed by immunocytochemistry (Supplementary Fig. 1–4).

## Discussion

At first presentation, both patients were diagnosed with CDI and their initial MRI abnormalities were a loss of hyperintense area on T1-weighted images (patients 1 and 2) or an ill-defined hypoenhanced area on gadolinium-enhanced MRI (patient 2). During treatment for CDI, anterior pituitary dysfunction developed, and sellar and suprasellar lesions became enlarged. Both patients were negative for anti-rabphilin-3A antibody, which suggested the exclusion of LINH. Finally, patients 1 and 2 were diagnosed with pure germinomas 15 and 14 months after a diagnosis of CDI, respectively, and were successfully treated.

Germinoma has the potential for rapid growth and dissemination; on the other hand, it is highly sensitive to radiation therapy and chemotherapy [22, 23]. Therefore, germinoma is an important differential diagnosis for CDI in children and young adults. However, the diagnosis of neurohypophyseal GCTs, including germinoma, is sometimes challenging. Endocrinopathies, including CDI, are not always accompanied by radiographic changes in the neurohypophysis; that is a small “occult” tumor is capable of disturbing hormone production [15]. Therefore, initial MRIs of these cases show only the loss of a hyperintense signal in the posterior hypophysis or equivocal thickened pituitary stalk [14, 24, 25]. Di Iorgi et al. prospectively observed idiopathic CDI in children or young adult patients with a normal or thickened pituitary stalk and found seven germinoma patients among 61 patients with idiopathic CDI within 2.5 years of follow-up [8]. Another report of seven CDI patients, ranging from 6 to 16 years of age, with a thickened pituitary stalk described five patients (71%) who were diagnosed with germinoma [26]. Takami et al. also reported that among 14 patients with neurohypophyseal GCT and CDI as an initial symptom, seven underwent MRI within 1 year of the onset of CDI symptoms of which five MRI findings were normal [11]. Subsequent neuroimaging ultimately diagnosed neurohypophyseal tumors in all individuals; however, the latency periods were 150, 315, 889, 942, and 1366 days. They also found that compared with patients without CDI as an initial symptom, the latency period from symptomatic onset to diagnosis was significantly longer among patients who developed early CDI (1083 vs. 360 days, *p* = 0.009) [11]. These data and our cases highlight the fact that germinoma should be suspected in all children and young adult patients with CDI. Therefore, it is recommended that a brain MRI be performed in patients with CDI of no clear etiology every 3–6 months for at least 5 years after initial presentation [12]. In our first case, serial MRI was performed and a gradual thickening of the pituitary stalk was observed, which led us to perform a biopsy. In contrast, in our second case, a follow-up MRI was not performed. When other symptoms in addition to CDI developed, the patient’s tumor had already extended to the suprasellar region. If a follow-up MRI had been performed, a biopsy and diagnosis of the germinoma might have been done earlier, possibly before the development of anterior pituitary dysfunction.

Both our cases developed anterior pituitary dysfunction more than 1 year after the diagnosis of CDI. Robison et al. reported that the co-occurrence of CDI and anterior pituitary dysfunction, either at presentation or on follow-up, was significantly associated with a subsequent diagnosis of the neoplastic process. In this report, seven of 16 patients with CDI and pituitary stalk thickening also showed anterior pituitary dysfunction, and six of these seven patients (86%) were ultimately found to have a neoplastic process in the pituitary stalk [27]. In another study, during the follow-up of patients without a known etiology for their CDI on initial evaluation and with no additional hypothalamic/pituitary deficiencies, the likelihood of an etiologic diagnosis increased from 20 to 50% when any additional endocrine deficiency (particularly GH deficiency) was diagnosed [7]. Therefore, these reports and our cases suggest that the underlying etiology for the CDI must be investigated, especially in the presence of anterior pituitary hormone deficiencies that increase the suspicion of an occult hypothalamic lesion [12, 28].

In patient 1, the PLAP concentration of the CSF was elevated. PLAP is a protein found on the surface of germinoma cells [29]. Shinoda et al. first found PLAP concentration to be increased in the serum and/or CSF of patients with primary intracranial germinomas [30]. Watanabe et al. reported that concentrations of CSF PLAP in germinoma cases were significantly elevated (median CSF PLAP level 425 pg/mL, range 16 to 3700 pg/mL, *n* = 36) and the sensitivity and specificity of a PLAP assay for germinomas were 94 and 97%, respectively, with a cut-off value of 30 pg/mL [21]. They concluded that in the case of suprasellar and pineal region tumors with normal values for the tumor markers, β-hCG and AFP, in serum and elevated PLAP in CSF, the only possible diagnosis is a germinoma. They subsequently speculated that under such circumstances, no histological analysis was required to confirm the diagnosis [21]. Though further research is required to corroborate the finding that germinoma can be diagnosed based on elevated CSF PLAP without biopsy, a negative test for anti-rabphilin-3A antibody would help this diagnostic process to exclude LINH.

We have previously reported that anti-rabphilin-3A antibody is a useful diagnostic marker for LINH [2, 20]. Anti-rabphilin-3A antibodies were detected in 22 of 29 patients (76%) (including four out of four biopsy-proven samples) with LINH. Anti-rabphilin-3A antibodies were not detected in all 34 biopsy-proven samples from sellar/suprasellar masses not due to lymphocytic hypophysitis, including five germinomas [20]. Recently, we reported that among 15 consecutive patients with CDI, anti-rabphilin-3A antibodies were found in four of five LINH cases and three of four lymphocytic panhypophysitis (LPH) cases. In addition, anti-rabphilin-3A antibodies were found in one germinoma of four biopsy-proven cases with sellar/suprasellar masses, including one germinoma, one craniopharyngioma, and two Rathke cleft cysts [18]. The presence of anti-rabphilin-3A antibodies was first demonstrated in a patient with a biopsy-proven germinoma [18]. The reason for the presence of anti-rabphilin-3A antibodies in this patient is unclear. However, it is possible that lymphocytes reactive to rabphilin-3A that infiltrated the posterior pituitary may play a role. Therefore, to date, among 38 biopsy-proven samples from sellar/suprasellar masses not due to lymphocytic hypophysitis, anti-rabphilin-3A antibodies have been reported in only one case. Case reports with respect to anti-rabphilin-3A antibodies are summarized as follows. In childhood, anti-rabphilin-3A antibodies were found in a 10-year-old boy [31] and 7-year-old boy [32] with LINH. Anti-rabphilin-3A antibodies were detected in patients with LINH-associated pregnancy [33] or diabetic ketoacidosis [34]. In addition, anti-rabphilin-3A antibodies were found in CDI patients with biopsy-proven LINH [35] or LPH [36]. Therefore, anti-rabphilin-3A antibodies are relatively common in LINH or lymphocytic hypophysitis, and may be valuable for differentiating between CDI etiologies.

In addition, rabphilin-3A may be a pathogenic antigen and T cells specific for rabphilin-3A may be involved in the pathogenesis of neurohypophysitis in mice [37]. Rabphilin-3A has been identified as an effector for RAB3A, which belongs to the small G protein superfamily and is thought to be a critical regulator of secretory vesicle trafficking, including in exocytosis [38]. Rabphilin-3A is expressed mainly in the brain and is reportedly involved in neurotransmitter release and synaptic vesicle traffic [39]. We have reported that rabphilin-3A is expressed in the posterior pituitary and AVP neurons in the hypothalamus and that rabphilin-3A is not expressed in the anterior pituitary [20]. We previously reported that immunization with rabphilin-3A led to neurohypophysitis in a mouse model. We also found that abatacept, which suppresses T cell activation, ameliorated lymphocytic infiltration of CD3 + T cells in the neurohypophysis and restored urine volume in mice immunized with rabphilin-3A [37]. These results suggest the proliferation and/or infiltration of autoreactive lymphocytes against rabphilin-3A in the neurohypophysis may be involved in the pathogenesis of LINH, which shows lymphocyte infiltration and AVP deficiency. Therefore, one of the reasons why anti-rabphilin-3A antibodies are detected in patients with LINH, but not in patients with germinoma and other tumors, could be that abnormalities in immunocompetent cells, such as lymphocytes that infiltrate the neurohypophysis and react to rabphilin-3A, may be involved in the pathogenesis of LINH.

Endocrine function often does not recover even after the disappearance of the germinoma, which can be on account of the effects of direct tumor growth or side effects due to radiotherapy and surgery [16, 40]. However, in our second case, pretreatment hypogonadism and a low IGF-1 concentration both recovered after chemoradiotherapy. Saeki et al. reported that none of 12 and four cases of impaired GH or TSH secretion, respectively, in the pretreatment of germinoma improved after treatment. They also reported only two of 12 impaired LH/FSH secretion cases and one of six impaired ACTH secretion cases improved after treatment [41]. In another study of germinoma, gonadotropin secretion improved in only two of 16 cases, with no improvement in 17 cases of impaired TSH and GH secretion [42]. One of the predictive factors of these rare events was reported to be a smaller tumor, more specifically, neurohypophyseal germinomas extending below the optic chiasm or localized to the pituitary stalk and the third ventricle floor [40]. We speculate that another predictive factor is older age, since prior cases of improved pituitary functions were in 17- [43], 18- [40], 20- [40], and 21- [44] year-old patients, while our second case was 21 years old; these are relatively older ages for germinoma patients. In adult survivors of childhood cancers, anterior pituitary dysfunction is less likely at an older age [45]. It is possible that a developed hypothalamic-pituitary system is more resistant to tumor invasion than a developing one.

In conclusion, it is very important to recognize that germinoma may occur in cases that show no or little abnormality on MRI at the onset of CDI in children and young adults. Therefore, regular MRI is strongly recommended in all patients with CDI of unknown etiology, especially in patients without measurable anti-rabphilin-3A antibodies. Furthermore, a negative test for anti-rabphilin-3A antibody, a useful marker of LINH, elevates the risk for the development of non-lymphocytic lesions, including germinoma in young patients with CDI.

### Supplementary Information

Below is the link to the electronic supplementary material.

Supplementary file1 (PPTX 19193 KB)

## Data Availability

The data that support the findings of this study are available on request from the corresponding author, YS. The data are not publicly available due to their containing information that could compromise the privacy of research participants.
