# Supplementary material for: Absence of anti–rabphilin-3A antibodies in children and young adults with idiopathic central diabetes insipidus: a potential clue to elucidating a tumor etiology
Source: Hormones (Athens). 2023 Sep 11;22(4):747–58. doi: 10.1007/s42000-023-00484-0 (PMC10651553; doi:10.1007/s42000-023-00484-0)
Supplement: Supplementary file 1 — Supplementary file1 (PPTX 19193 KB) [file 42000_2023_484_MOESM1_ESM.pptx]

## Slide 1
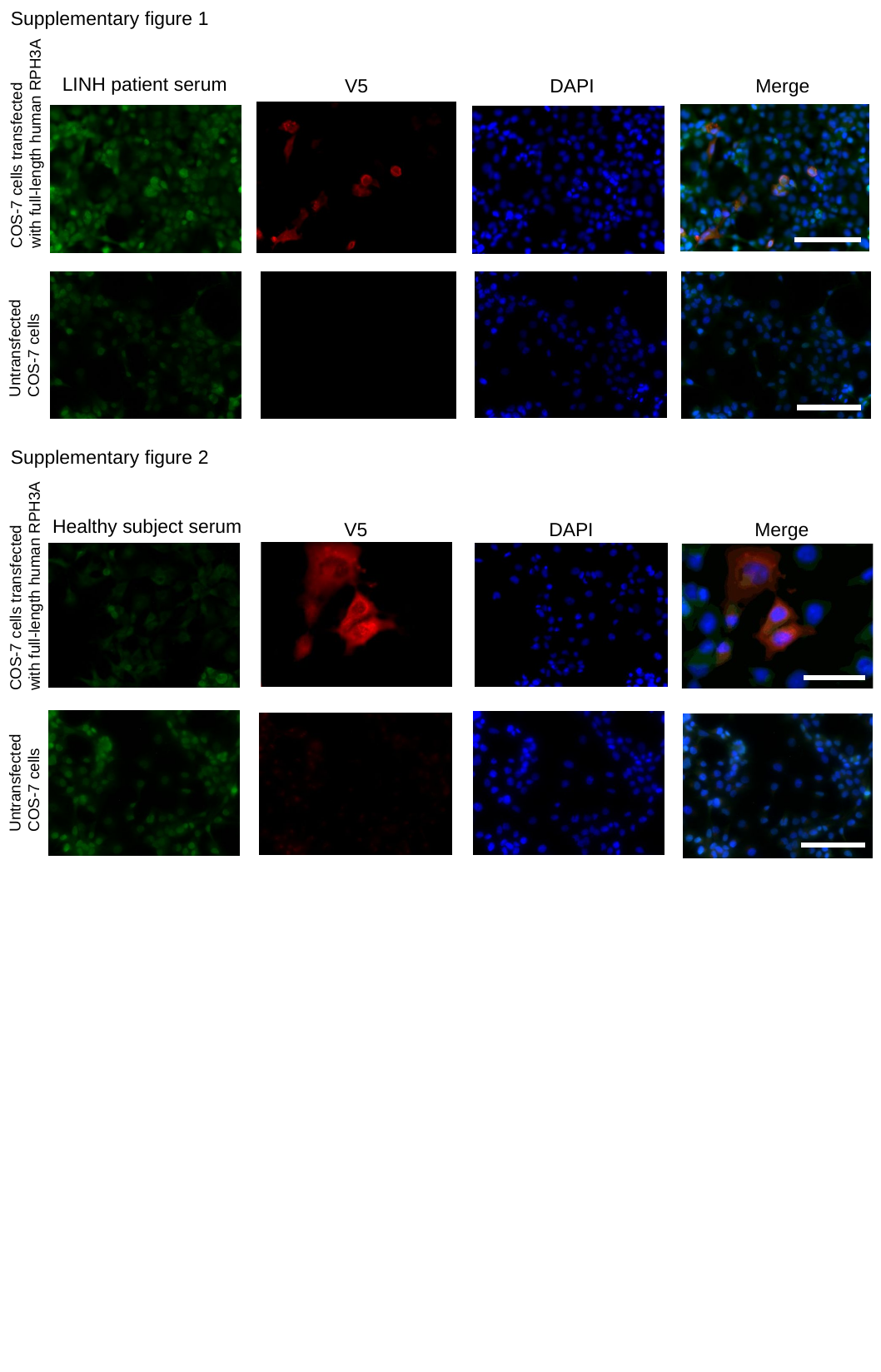

Supplementary figure 1
LINH patient serum
DAPI
Merge
V5
COS-7 cells transfected
with full-length human RPH3A
Untransfected
COS-7 cells
Supplementary figure 2
Healthy subject serum
DAPI
Merge
V5
COS-7 cells transfected
with full-length human RPH3A
Untransfected
COS-7 cells

## Slide 2
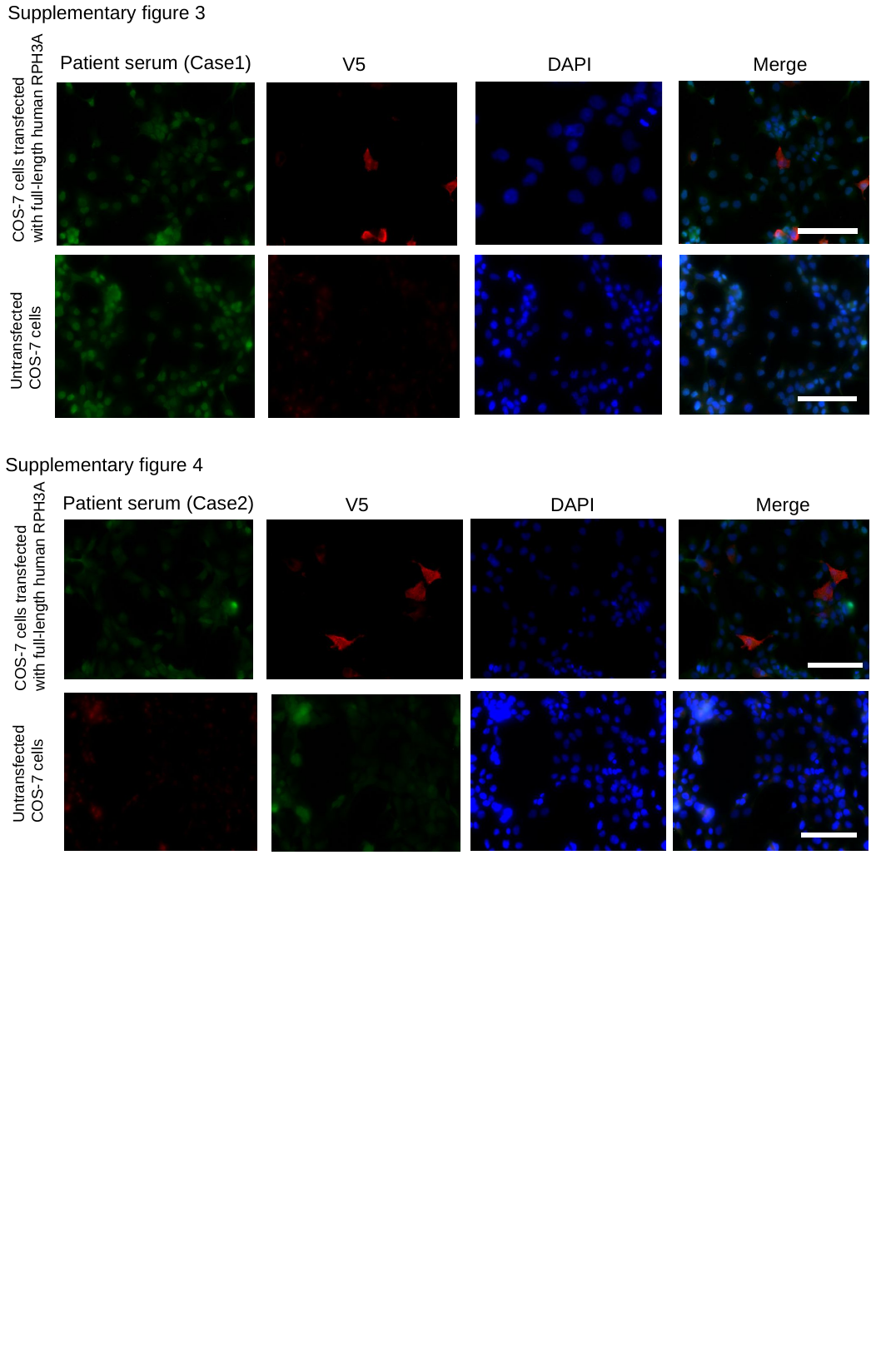

Supplementary figure 3
Patient serum (Case1)
DAPI
Merge
V5
COS-7 cells transfected
with full-length human RPH3A
Untransfected
COS-7 cells
Supplementary figure 4
Patient serum (Case2)
DAPI
Merge
V5
COS-7 cells transfected
with full-length human RPH3A
Untransfected
COS-7 cells
